# Emerging roles of pyruvate dehydrogenase phosphatase 1: a key player in metabolic health

**DOI:** 10.3389/fphys.2025.1596636

**Published:** 2025-05-26

**Authors:** Vikalp Kumar, Miriam L. Greenberg

**Affiliations:** Department of Biological Sciences, Wayne State University, Detroit, MI, United States

**Keywords:** pyruvate dehydrogenase complex, pyruvate dehydrogenase phosphatase 1, cancer, traumatic brain injury, cardiomyogenesis, barth syndrome

## Abstract

Pyruvate dehydrogenase phosphatase (PDP), a structurally conserved member of the protein phosphatase C family (PP2C) of proteins, is a key regulatory enzyme responsible for reactivation of the mitochondrial gate-keeper, pyruvate dehydrogenase (PDH). Tissue-specific expression of PDP isozymes, specifically PDP1 and PDP2 facilitate regulation of the multi-subunit PDH, influencing flux of substrates to the TCA cycle. PDP1 is a heterodimeric, Ca^2+^ sensitive isoform, predominantly expressed in muscle tissue where its role in regulating PDH activity is well established. Emerging research suggests that it is involved in various diseases, including pancreatic ductal adenocarcinoma, cardiomyogenesis defects, traumatic brain injury, and Barth syndrome. In this review, we discuss recent studies revealing the crucial role of PDP1 and its dysregulation in various metabolic disorders, thereby highlighting its potential as a therapeutic target for these debilitating diseases.

## 1 Introduction

Cellular energy homeostasis hinges on the intricate balance of ATP levels. Regulation of ATP, the cellular currency of free energy, has important ramifications for maintaining cell physiology and metabolism. Under normal physiological conditions, oxidative metabolism is a major driver of ATP production constituting a series of metabolic reactions mediated by enzymes in the cytosol and in mitochondria of eukaryotic cells (Vanderperre et al., 2015; Wilson, 2017). Accordingly, a glucose molecule is metabolized in the cytoplasm via glycolysis, yielding two molecules of pyruvate. Pyruvate may be fermented in the cytoplasm -to lactate by lactate dehydrogenase (LDH), replenishing NAD^+^ pools essential for sustaining glycolysis. Alternatively, pyruvate may be converted to acetyl-CoA and utilized in ATP production via the TCA cycle and oxidative phosphorylation in the mitochondria (Gray et al., 2014; Vanderperre et al., 2015).

**FIGURE 1:**
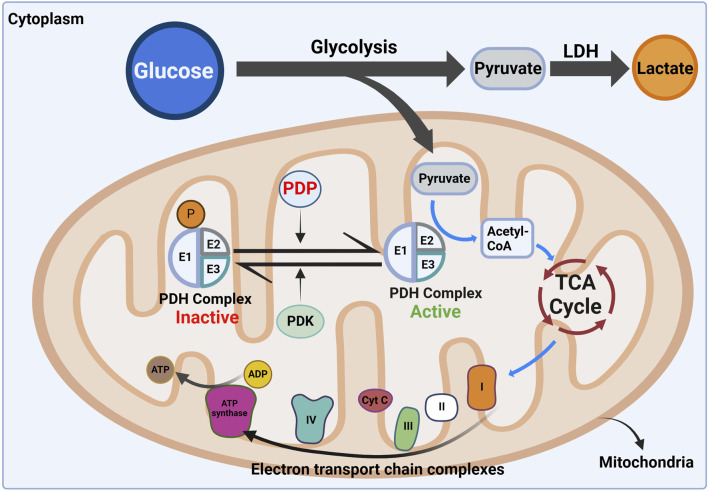
Role of pyruvate dehydrogenase complex (PDH) in cellular energy metabolism. The diagram shows the metabolic fate of glucose-derived pyruvate in the cytoplasm and mitochondria. Glycolysis involves the breakdown of glucose into pyruvate, which can either be converted to lactate by lactate dehydrogenase (LDH) or transported into mitochondria. Within the mitochondria, the PDH converts the pyruvate to acetyl-CoA, feeding into the tricarboxylic acid (TCA) cycle for energy production. PDH activity is regulated by reversible phosphorylation: Pyruvate dehydrogenase kinase (PDK) inactivates it, while pyruvate dehydrogenase phosphatase (PDP) reactivates it, controlling metabolic flux to the TCA cycle. Acetyl-CoA enters the TCA cycle, fueling ATP synthesis via oxidative phosphorylation in the ETC, crucial for glucose oxidation and energy homeostasis. Figure Source: Created in BioRender. Vo, L. (2025) https://BioRender.com/n28p718.

Pyruvate molecules enter mitochondria through the mitochondrial pyruvate carrier (MPC) and are predominantly processed by the multicomponent machinery of the PDH complex referred to as PDC or PDH in the literature. The PDH complex catalyzes decarboxylation of pyruvate into the two-carbon molecule, acetyl CoA, which subsequently feeds into the tricarboxylic acid (TCA) cycle (Patel and Roche, 1990; Harris et al., 2002; Gray et al., 2014; Patel et al., 2014; Vanderperre et al., 2015). Fine tuning of PDH activity mainly occurs by covalent modifications and allosteric regulation. Interestingly, covalent modification occurs only in eukaryotes and is mediated by the tightly associated PDH kinase (PDK) and PDH phosphatase (PDP), which catalyzes reversible phosphorylation and dephosphorylation of PDH, respectively (Harris et al., 2002; Byron and Lindsay, 2017; Park et al., 2018; Karagiota et al., 2023) (Figure 1). Regulation of PDH is crucial to maintain cellular energy balance and to adapt to varying physiological conditions, including pyruvate levels, the acetyl-CoA/CoA ratio, and the NAD^+^/NADH balance (Holness and Sugden, 2003).

While the function of PDH has been widely studied and reviewed (Patel and Roche, 1990; Harris et al., 2002; Holness and Sugden, 2003; Patel et al., 2014; Byron and Lindsay, 2017; Park et al., 2018), much less is known about PDP1. In this review, we explore key mechanisms underlying the regulation of PDH complex, followed by the molecular characteristics, structure, and function of PDP1. Lastly, we delve into the health implications of PDP1 and its potential as a target for therapeutic interventions.

## 2 PDH complex and its regulation

PDH is a multienzyme complex that plays a crucial role in cellular respiration (Patel and Roche, 1990; Gray et al., 2014; Lee, 2014; Patel et al., 2014; Vanderperre et al., 2015). It is present in the mitochondria of eukaryotic cells and in the cytosol of prokaryotes. The complex is composed of three catalytic subunits, pyruvate dehydrogenase (E1), dihydrolipoamide transacetylase (E2), and dihydrolipoamide dehydrogenase (E3). The oxidation of pyruvate to acetyl-CoA by E1, E2, and E3 requires coenzymes thiamine pyrophosphate (TPP), lipoic acid, coenzyme A (CoA), FAD^+^, and NAD^+^ (Patel et al., 2014; Byron and Lindsay, 2017). In higher eukaryotes, the PDH complex contains additional components, including dihydrolipoamide dehydrogenase binding protein (E3BP) and two regulatory enzymes, PDH kinase (PDK) and PDH phosphatase (PDP) (Holness and Sugden, 2003; Lee, 2014; Patel et al., 2014; Byron and Lindsay, 2017; Park et al., 2018).

The activity of PDH complex is regulated mainly by two key processes, allosteric inhibition and covalent modification (Sheeran et al., 2019). Allosteric regulation of PDH complex is controlled by NADH and acetyl-CoA levels. High [NADH]/[NAD^+^] and [acetyl-CoA]/[CoA] ratios result in allosteric inhibition of PDH complex (Holness and Sugden, 2003). Conversely, a decrease in these ratios results in the activation of PDH complex allosterically (Pettit et al., 1975; Spriet and Heigenhauser, 2002; Holness and Sugden, 2003).

Regulation of PDH activity via covalent modification is uniquely restricted to the eukarya domain. This process involves cyclic phosphorylation and dephosphorylation events, facilitated by tightly associated PDKs and PDPs, respectively (Holness and Sugden, 2003; Byron and Lindsay, 2017; Park et al., 2018; Karagiota et al., 2023; Yang et al., 2024). The phosphorylation of one or more serine residues (Ser 264, Ser 271, and Ser 203) on the α-chain of enzyme E1, catalyzed by PDKs, leads to inactivation of the complex (Holness and Sugden, 2003). However, phosphorylation can be reversed by two different isozymes of PDPs, PDP1 and PDP2, resulting in the reactivation of the E1 subunit of the PDH complex (Korotchkina and Patel, 1995; Kolobova et al., 2001; Korotchkina and Patel, 2001; Roche et al., 2001; Kato et al., 2008; Byron and Lindsay, 2017). The expression of PDP isoforms is tissue-specific. PDP1 is predominantly expressed in muscles, while liver and adipose tissue mainly express the PDP2 isoform (Byron and Lindsay, 2017). Notably, PDP1 is distinctively sensitive to Ca^2+^ and Mg^2+^, allowing it to respond rapidly to muscle specific metabolic demands (Holness and Sugden, 2003). During muscle excitation-contraction coupling Ca^2+^ released from the sarcoplasmic reticulum activates PDP1 within mitochondria, followed by dephosphorylation and reactivation of PDH (Denton et al., 1972). In contrast to PDP1, PDP2 is less sensitive to Ca^2+^ and Mg^2+^ ions and is activated by biological polyamine spermine. While alterations in PDP1 expression or activity typically result in muscle and neurological pathologies, the dysregulation of PDP2 is more associated with liver-specific metabolic disorders, such as non-alcoholic fatty liver disease and insulin resistance (Shannon et al., 2021). While both PDP1 and PDP2 are involved in PDH activation, PDP1 more substantially impacts PDH activity and thus has been more directly linked to metabolic diseases (Table 1). This underscores the necessity of understanding the role of PDP1 in metabolic health and diseases.

**TABLE 1:** Comparative overview of PDP1 and PDP2. The table summarizes key differences between PDP1 and PDP2, including their expression patterns across tissues, regulatory features, and known associations with disease states such as cancer, traumatic brain injury (TBI), cardiomyogenesis defects, and Barth syndrome.

Features	PDP1	PDP2
Tissue distribution	Ubiquitously expressed, with higher levels in energy-demanding tissues such as the skeletal muscles, heart, and brain	Predominantly expressed in the liver, adipose tissue with lower levels in other tissues
Regulation	Activated by Mg2+ and Ca2+ ions and subject to transcriptional regulation in response to metabolic demands	Less sensitive to Mg2+ and Ca2+ ions. In contrast to PDP1, it is activated by polyamine spermine. Its regulatory mechanisms are less well-characterized but may be regulated by nutritional states and insulin signaling
Diseases		
Cancer	Overexpression linked to enhanced proliferation, invasion, and migration in various cancers, including pancreatic ductal adenocarcinoma, leukemia, and colorectal cancers	Not significantly implicated in cancer progression
Traumatic Brain Injury (TBI)	Region- specific alterations in the expression levels Post- TBI. Potential involvement in neuronal cell death, and thalamic energy metabolism	Lack of evidence to support direct links to TBI
Cardiomyogenesis defects	Altered expression patterns regulate differentiation process of embryonic stem cells to cardiac myocytes	Role in cardiomyogenesis is not well-defined
Barth syndrome	Impaired PDP1 activity observed in Barth syndrome models, leading to reduced pyruvate dehydrogenase activity and compromised mitochondrial function	No direct association established with Barth syndrome

## 3 Molecular characterization of PDP1

PDP1 is a heterodimeric protein consisting of a 50 kDa catalytic subunit (PDP1c) and a 97 kDa regulatory subunit (PDP1r) (Roche et al., 2003; Kato and Kato, 2010). The PDP1c subunit requires bivalent cations (Mg^2+/^Mn^2+^) to catalyze the dephosphorylation of PDH. PDP1r is a flavin-dependent regulatory subunit with poorly understood function (Vassylyev and Symersky, 2007; Kato and Kato, 2010; Guo et al., 2020). Interestingly, the activity of PDP1c is stimulated by Ca^2+^, which could explain the predominance of this isoform in muscle tissues (Vassylyev and Symersky, 2007; Kato and Kato, 2010). Ca^2+^ facilitates the binding of PDP1c to the L2 (inner lipoyl) domain within the E2 subunit of the PDH complex, thus increasing the dephosphorylation activity of PDP1 and the reactivation of PDH (Turkan et al., 2002; Turkan et al., 2004; Kato and Kato, 2010; Guo et al., 2020). PDP1c shares very low sequence identity (21%–24%) with the Mg^2+^ dependent protein phosphatase C family (PP2C) members of Ser/Thr phosphatases. However, the structural features of α-helices and loops surrounding the central β-sandwich are highly conserved among PDP1c and the members of the PP2C family of proteins (Vassylyev and Symersky, 2007). Notably, human PDP1c shares 98% sequence identity with the rat PDP1c (Kato and Kato, 2010).

Analysis of the PDP1c crystal structure from rat reveals that the protein comprises two nearly identical molecules linked by rotational symmetry (Vassylyev and Symersky, 2007). Each molecule comprised of two anti-parallel β-sheets flanked by α-helices and disordered regions (loops) on each side (Vassylyev and Symersky, 2007). A distinctive aspect of PDP1c is the presence of a unique hydrophobic pocket on the surface (residues 98–151), which probably interacts with the lipoyl moiety of the E2 subunit of the PDH complex (Vassylyev and Symersky, 2007). In contrast, bovine PDP1c is a single molecule with a core of β-sheets, which is encircled by α-helices connected by surface loops (Guo et al., 2020). In an aqueous environment, PDP1c exists in a dynamic equilibrium between monomeric and dimeric states, which can alter the generation of functional complexes with other proteins (Turkan et al., 2004; Vassylyev and Symersky, 2007). The catalytic site of PDP1c is distinguished by a binuclear cluster of magnesium atoms (manganese atoms in the case of bovine PDP1c), and the dimer observed in crystallography studies may correspond to the dimeric state observed in solution (Vassylyev and Symersky, 2007; Kato and Kato, 2010; Guo et al., 2020). Dimerization of PDP1c stabilizes the catalytic conformation compared to the monomeric form, enhancing its phosphatase activity towards PDH. The availability of calcium and magnesium ions modulates the monomer-dimer equilibrium dynamics. The presence of PDP1r can alter or obstruct the catalytic site of PDP1c, potentially mediating the regulation of PDP1c activity (Vassylyev and Symersky, 2007). Importantly, disruptions in PDP1 activity affecting its structural states or catalytic site configuration can directly impact the function of PDH complex. This dysfunction may contribute to metabolic imbalances such as those observed in Barth syndrome (Liang et al., 2024) and various neurodegenerative disorders (Sorbi et al., 1983).

## 4 Implications for human health

PDH is a pivotal gate keeper of energy metabolism, funneling pyruvate molecules from glycolysis into the TCA cycle in the form of acetyl CoA, a vital intermediate of oxidative metabolism. In light of the pivotal role of PDH in energy metabolism, as discussed above, it is not surprising that disruption of the PDH-PDP1 axis contributes significantly to metabolic dysregulation, as observed in cancer, neurodegenerative, and other metabolic disorders (Sorbi et al., 1983; Shi et al., 2021; Liang et al., 2024).

### 4.1 PDP1 and cancer metabolism

#### 4.1.1 Leukemia

The growth of malignant cells relies heavily on glycolysis for energy production, but the molecular mechanisms underlying the metabolic switch from oxidative phosphorylation to glycolysis during malignancy are not well-understood. Shan et al. reported that phosphorylation of PDP1 by various oncogenic tyrosine kinases, such as FGFR1, ABL, and JAK2, inhibit PDP1 activity by reducing its binding affinity to lipoic acid. This leads to enhanced inhibition of PDH, thereby promoting glycolysis over oxidative phosphorylation in neoplastic cells (Shan et al., 2014). Interestingly, they observed that phosphorylation of PDP1 at Tyr-94 is common in various human tumor cells, e.g., A549 lung cancer cells and MDA-MB-231 breast cancer cells. This study found elevated expression of phosphorylated Tyr-94 in the primary leukemia cells isolated from leukemia patients compared to normal cells from healthy individuals (Shan et al., 2014) suggesting a correlation between tyrosine phosphorylation of PDP1 and the cancer pathophysiology.

Studies identified PDP1 as a modulator of drug resistance in the Fms-like tyrosine kinase 3- internal tandem duplications (FLT3-ITDs) positive leukemia (Alshamleh et al., 2023). PDP1 knockdown resulted in decreased respiration in FLT3-ITDs positive cells, thereby affecting the proliferation rates (Alshamleh et al., 2023). PDP1 may thus be a potential therapeutic target to revert the sensitivity of drug resistant tumor cells and leukemia treatment.

#### 4.1.2 Pancreatic ductal adenocarcinoma

Pancreatic cancer ranked eighth in mortality among all other cancers reported worldwide according to the Global Burden of Diseases, Injuries, and Risk Factors Study (GBD) (Collaborators, 2019). According to recent global estimates, the incidence of PDAC has doubled over the past 25 years. Projections indicate that within the next 20–30 years, PDAC is expected to become the second leading cause of cancer-related deaths in the United States (Mizrahi et al., 2020). Elevated expression of PDP1 has been reported in human pancreatic ductal adenocarcinoma (PDAC) and is correlated with poor prognosis of the disease (Li et al., 2020). PDAC is a life-threatening malignant condition associated with pancreatic glands. The findings by Li et al. underscore the involvement of AMPK/mTOR signaling in PDP1 regulation and PDAC advancement (Li et al., 2020). The study showed that increased PDP1 expression results in sustained suppression of AMPK signaling, subsequently facilitating mTOR and stimulating cancer cell proliferation, movement, and expansion (Li et al., 2020). Furthermore, the group demonstrated alternating PDP1 levels, either through stable PDP1 expression or shRNA-mediated knockdown, which directly leads to changes in AMPK and mTOR activity.

#### 4.1.3 Colorectal cancer

Colorectal cancer is among the most common cancers worldwide (Yuan et al., 2024). It is a malignant tumor that develops in the posterior regions of large intestine, colon and rectum. The tumor often arises from malignant transformation of polyps. Shi et al. found that the aberrant expression of PDP1, PDH, and mitochondrial electron transport chain Complex I is associated with a poor prognosis of colorectal cancer (Shi et al., 2021). Impairments in mitochondrial complex I activity led to reduced levels of PDH in both the cytoplasm and nucleus. Reduced nuclear localization of PDH results in decreased histone acetylation, enhancing the DNA damage repair response and conferring increased resistance to radiation, highlighting the importance of targeting the [Ca^2+^]-PDP1-PDH retrograde signaling axis to improve the effectiveness of radiotherapy in colorectal cancer patients (Shi et al., 2021). The findings by Yuan et al. underscore the increased expression of PDP1 mediated by the transcription factor KLF5 in KRAS mutant colorectal cancer cells and tissue compared to wild-type, correlates with a poor prognosis of colorectal cancer. The study shows that PDP1 enhances BRAF and MEK1 interaction and activates MAPK signaling, thereby promoting cancer progression (Yuan et al., 2024).

In summary, PDP1 plays an important role in cancer metabolism by controlling the shift between oxidative phosphorylation and glycolysis, a key characteristic of cancer progression. Studies showing phosphorylation of PDP1 at Tyr-94 in various tumor cells, elevated PDP1 expression resulting in the suppression of AMPK signaling in PDAC, and aberrant expression in colorectal cancer, enhanced inhibition of PDH activity and promoting tumor progression, underscore PDP1 as a key metabolic regulator in cancer, proposing it as a promising target for diagnosis and improved treatment outcomes.

### 4.2 PDP1 and traumatic brain injury

Traumatic brain injury (TBI) is characterized by several metabolic disturbances including lactic acidosis, perturbed glucose metabolism, and pyruvate depletion, which lead to secondary neuronal damage and impaired recovery (Buczek et al., 2002; Hattori et al., 2003; Xing et al., 2012). The role of PDP1 in metabolic disruptions during TBI emerged from the study by Xing et al., who observed region-specific alterations in the expression of PDP1 mRNA (Xing et al., 2012). These include significant downregulation in cerebral cortex and hippocampus, but upregulation in the thalamic regions within the central nervous system post TBI, suggesting that differential expression of PDP1 may lead to hypoglycemia in the cortex and hippocampus and hyperglycemia in the thalamus. This finding underscores the potential role of PDP1 as a transcriptional marker for TBI-induced metabolic perturbations (Xing et al., 2012).

Notably, PDP1 expression appears to be regulated in a differential manner across distinct brain regions, particularly within hippocampus, cerebral cortex, and thalamic regions during the acute phase of TBI (Xing et al., 2012). This finding aligns with other studies that have shown region-specific metabolic rewiring in glucose utilization and oxidative phosphorylation post TBI (Buczek et al., 2002; Lifshitz et al., 2003). Furthermore, implication of PDP1 in neuronal cell death, with significant impacts on thalamic energy metabolism and impaired recovery post-injury (Xing et al., 2012), highlights the potential of PDP1 as a therapeutic target for alleviating metabolic dysfunctions associated with TBI.

### 4.3 PDP1 deficiency

A deficiency of PDP1 has been reported to cause severe exercise intolerance, lactic acidosis, and mild developmental delay. Maj et al. studied two brothers of consanguineous parents presenting clinical symptoms of neonatal hypotonia and elevated lactate levels concomitant with diminished PDH activity in skin fibroblasts (Maj et al., 2005). The cause of the defect was mapped to a 3bp deletion in the coding sequence of PDP1, eliminating a leucine residue from the 213 position of the protein (Maj et al., 2005; Maj et al., 2006; Cameron et al., 2009). Furthermore, Cameron et al. identified a null mutation in PDP1 as an underlying cause of lethal infantile phenotype (Cameron et al., 2009). The group reported a homozygous mutation within the PDP1 gene in the fibroblast of a female patient showing symptoms of lactic acidosis and mild truncal hypotonia. The mutation was mapped to a guanine to thymine substitution (c.277G > T (p.E93X), creating a premature stop codon. Consequently, the truncated protein resulted in early stage fatality due to acute respiratory distress (Cameron et al., 2009). These findings underscore the indispensable role of PDP1 in maintaining metabolic homeostasis and respiratory stability.

### 4.4 PDP1 and cardiomyocyte differentiation

Embryonic stem cells (ESCs) with self-renewal and pluripotent properties are an attractive model system for differentiation processes (Thomson et al., 1998; Keller, 2005; Kim et al., 2023). The cells can give rise to various cell lineages on differentiation and have been a highly manipulated tool to study early cardiomyogenesis (Boheler et al., 2002; Kumar et al., 2005; Lev et al., 2005). Several molecular factors, including cardiac-specific transcription factors and hypoxic environment, have been attributed to the differentiation process of ESCs into cardiomyocytes (Horton and Auguste, 2012; Turbendian et al., 2013). Recently, PDP1 has been reported as a potential regulator of the differentiation of early ESCs into cardiomyocytes (Heo et al., 2016). Heo et al. showed a decrease in PDP1 expression levels (27-fold) from day 0 to day 8 of the differentiation process of ESCs into cardiac myocytes, accompanied by decreased mitochondrial activity. Furthermore, the group showed that overexpression of PDP1 restored mitochondrial activity but diminished expression of the cardiac differentiation marker suggesting that PDP1 is a potential regulator of the differentiation process of ESCs into cardiomyocytes (Heo et al., 2016). Mechanistically, PDP1 crosstalk with hypoxia-inducing factor α (HIF1α) and reactive oxygen species (ROS) signaling pathways, which are involved in the metabolic reprogramming of ESCs during differentiation into cardiomyocytes. Hypoxic condition, often present in differentiating embryoid bodies, activates HIF1α, which upregulates PDK1 expression. Increased PDK1 levels enhance PDH’s inactivation, reducing mitochondrial oxygen consumption and ROS generation. PDP1 expression influences this HIF1α driven metabolic adaptation. Decreasing the levels of PDP1 during differentiation of ESCs into cardiomyocytes favors maintaining lower PDH activity and mitochondrial function, facilitating cardiomyocyte differentiation. These findings underscore PDP1 as a key metabolic regulator during cardiomyogenesis.

### 4.5 PDP1 and Barth syndrome

Barth syndrome (BTHS) is a life-threatening genetic disorder caused by a mutation in the TAFAZZIN gene (*TAZ)*, encoding a transacylase involved in the remodeling of the mitochondrial phospholipid-cardiolipin (CL) (Vreken et al., 2000; Saric et al., 2015; Ghosh et al., 2019; Chin and Conway, 2020; Kagan et al., 2023; Vo et al., 2023). PDP1 activity is significantly downregulated in the BTHS mouse myoblast model, as shown by Liang et al. (Liang et al., 2024). The group showed decreased PDP1 activity, concomitant with decreased PDH activity, resulting in diminished mitochondrial functionality in tafazzin-knockout (TAZ-KO) C2C12 myoblast cells, a cellular model of BTHS. The decrease in PDP1 activity is partly attributed to reduced mitochondrial calcium levels in TAZ-KO cells. Additionally, reduced interaction between PDP1 and PDH was observed in these cells concomitant with decreased PDP1 activity. Strikingly, the supplementation of solubilized mitochondria with tetralinoleoyl-CL (TLCL) restored its activity, leading to restoration of PDH function. TLCL is the predominant CL species in normal heart (Schlame et al., 1993; Sparagna et al., 2007; Shen et al., 2015; Semba et al., 2019) and is significantly reduced in *TAZ*-deficient cells, BTHS patients, and in TAZ cardiomyocyte-specific knockout mice (Zhu et al., 2021). Furthermore, the findings from this study suggest that TLCL may act as a scaffolding molecule, enhancing the interaction between PDH and PDP1 and thereby facilitating PDH activation. The group shows the rescue of PDP1 activity; concurrently, PDH activation is TLCL dose-dependent, wherein increasing TLCL levels appear to provide additional binding sites for PDP1 to interact with PDH. These findings underscore the significant role of PDP1 in mitochondrial energy metabolism, highlighting its potential implications for the treatment of BTHS.

## 5 Conclusion

**FIGURE 2:**
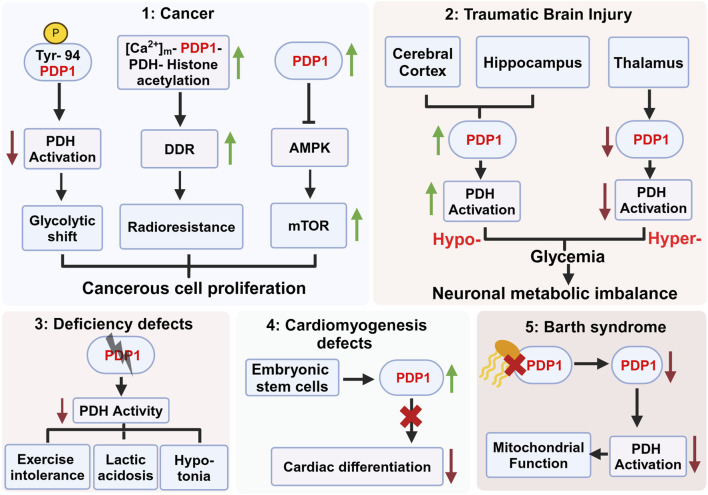
Role of pyruvate dehydrogenase phosphatase 1 (PDP1) in various pathophysiological conditions. This figure highlights the diverse roles of PDP1 in different diseases and physiological conditions. (**1**) PDP1 is implicated in cancer progression by modulating PDH activation, glycolytic shift, and radioresistance mediated by the activation of DNA damage repair (DDR) machinery. Increased PDP1 expression modulates AMPK-mTOR signaling, contributing to the fitness and proliferation of cancerous cells. (**2**) PDP1 expression varies across brain regions post Traumatic Brain Injury (TBI), with increased expression in the cerebral cortex possibly leading to hypoglycemia and decreased expression in the thalamus resulting in hyperglycemia. This imbalance contributes to neuronal metabolic dysregulation and survival. (**3**) PDP1 deficiency in deficiency defects result in decreased PDH activity, leading to exercise intolerance, lactic acidosis, and hypotonia. (**4**) In embryonic stem cells (ESCs), PDP1 deficiency impairs cardiac differentiation, leading to cardiomyogenesis defects, indicating its crucial role in heart development. (**5**) Decreased cardiolipin (yellow) binding to PDP1 in Barth syndrome compromises mitochondrial function, reducing PDH activation and leading to metabolic abnormalities. Figure Source: Created in BioRender. Vo, L. (2025) https://BioRender.com/x77q545.

Despite the well characterized role of PDP1 in the regulation of PDH and mitochondrial metabolism, its functional implications for human health and as a potential therapeutic target are only beginning to be understood. Emerging evidence points to critical involvement of PDP1 in various disorders (Figure 2), but specific mechanisms underlying its mode of regulation are not fully understood. The involvement of PDP1 in metabolic aberrations associated with devastating diseases underscores the necessity for more intensive investigation of the role of PDP1 in pathophysiological signaling underlying these devastating diseases. As an example, determining how PDP1 phosphorylation at Ty-94 leads to metabolic reprogramming in tumor cells may provide a deeper understanding of the role of PDP1 in tumor metabolism and drug resistance. Further, elucidating the molecular pathways that result in elevated expression of PDP1 in human PDAC may provide deeper insights into its correlation with poor prognosis of the disease. Moreover, deciphering signaling cascades leading to the region-specific expression of PDP1 mRNA in the central nervous system post-TBI may provide a deeper understanding of TBI-associated rewiring. Understanding how PDP1 regulates the differentiation of ESCs into cardiomyocytes may open a new avenue for cardiac regenerative therapies. Although no specific small-molecule activators or inhibitors of PDP1 are in clinical use to date, advancing our comprehension of its regulatory roles may pave the way for the development of pharmacologically active compounds capable of modulating its activity, thereby broadening its potential as a therapeutic target for these debilitating metabolic disorders.
